# Recycling Thermoset Epoxy Resin Using Alkyl-Methyl-Imidazolium Ionic Liquids as Green Solvents

**DOI:** 10.1021/acsapm.1c00896

**Published:** 2021-10-11

**Authors:** Rocío L. Pérez, Caitlan E. Ayala, Michelle M. Opiri, Abdulrahman Ezzir, Guoqiang Li, Isiah M. Warner

**Affiliations:** † Chemistry Department, Louisiana State University, Baton Rouge, Louisiana 70803, United States; ‡ Department of Mechanical & Industrial Engineering, Louisiana State University, Baton Rouge, Louisiana 70803, United States

**Keywords:** thermoset epoxy resin, carbon fiber reinforced composites, green recycling procedure, ionic liquids, recovery of recycled material

## Abstract

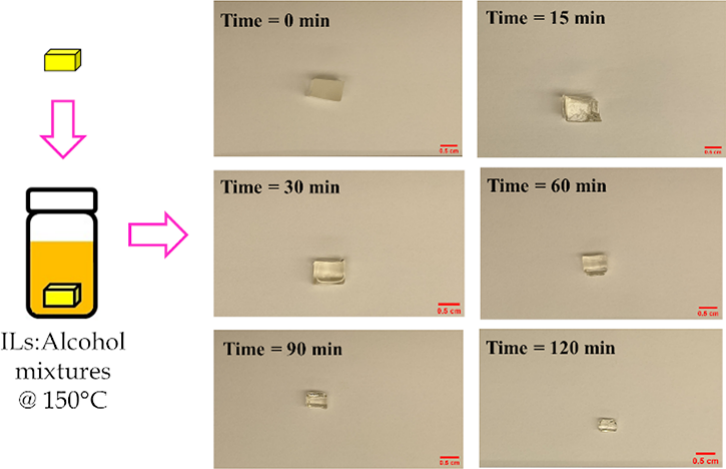

Herein, a solvent-based green recycling procedure is reported for recycling thermoset epoxy resins (TERs) and carbon fiber reinforced epoxy composites (CFRECs) employing ionic liquids (ILs) and alcohols under mild conditions. With melting points less than 100 °C, ILs are defined as organic salts, typically composed of bulky cations with organic or inorganic counteranions. As a result of their unique physical properties such as low vapor pressure, relatively high thermal stability, and multifunctional tunability, these solvents are often classified as “green solvents” as compared to traditional organic solvents. In this study, swelling and dissolution of TER are evaluated in the presence of pure alkyl-methyl-imidazolium ILs, alcohols, and various mixtures of these co-solvents to determine their swelling and depolymerization capacity at mild temperatures in the absence of catalysts. In these studies, three ILs with different alkyl lengths were evaluated: 1-butyl-3-methyl imidazolium chloride ([BMIm][Cl]), 1-hexyl-3-methyl imidazolium bromide ([HMIm][Br]), and 1-octyl-3-methyl imidazolium bromide ([OMIm][Br]) along with two alcohols: ethylene glycol (EG) and glycerol (Gly). The highest swelling capacity of TER at 150 °C was achieved by a combination of [BMIm][Cl] and EG. In addition, swelling and dissolution of TER were evaluated in the presence of several anion variants of 1-butyl-3-methyl-imidazolium ILs with EG. Complete dissolution of both TERs and CFRECs was achieved in 150 min (2.5 h) at 150 °C under atmospheric pressure. Finally, recovery and reuse of the recycled monomer after dissolution were examined. Recovered epoxy monomers employed to synthesize a recycled TER exhibited similar mechanical properties to the parent TER. In addition, it was demonstrated that carbon fibers could be successfully recovered from CFREC using the recycling method detailed in this manuscript.

## Introduction

Thermoset epoxy resins (TERs) are cross-linked polymers that exhibit excellent physical and mechanical properties, such as high chemical and solvent resistance, high glass transition temperature, excellent adhesion to substrates, high conductivity and impact resistance, and low thermal and solvent shrinkage.^1−4^ These characteristic properties make these materials attractive for use in various industrial application areas such as coatings, aerospace, automotive, sporting goods, electronic materials, adhesives, etc.^1−4^ For load carrying lightweight structures, carbon fiber reinforced epoxy composites (CFRECs) have been playing an important role.^5−7^ For these reasons, worldwide use of TER and CFREC materials has increased and is projected to grow from $17.5 billion in 2020 to $31.5 billion by 2025.^8^

Although the projected revenue from these types of materials has high potential, there is a high cost associated with common epoxy matrix and, particularly, with newly manufactured carbon fibers.^9^ Furthermore, the major consumption of these types of epoxy materials as well as the final manufactured products generates enormous waste, and most of these unwanted products are discarded into landfills.^10,11^ Thus, global consumption of such materials presents a major environmental concern and recovering these high-cost materials from waste would represent considerable economic benefit.^12−14^ For these reasons, different recycling methods have been investigated and employed to decrease waste associated with these types of materials.^10,15^

Mechanical and pyrolysis degradation methods are widely used to recycle TERs and CFRECs.^10,16^ These procedures are simple to scale up, making them a popular choice in industry.^9^ Nevertheless, both methodologies also present certain disadvantages, including higher consumption of energy, decreased quality, and distorted morphology of recycled carbon fibers (CF) as a result of grinding and deposition of charcoal over fibers during the pyrolysis process.^10,17,18^ As a result, considerable research is focused on developing methodologies to recycle TER and CFREC with the objective of recovering monomers from TER and recycling high-quality carbon fibers for further use. Several studies have been reported regarding use of supercritical solvents for recycling TER.^19−23^ For example, Okajima et al. have developed a recycling method for a carbon fiber reinforced polymer (CFRP), which employs superheat and supercritical acetone.^19^ Optimal conditions of operations established by these authors were 350 °C at 14 MPa pressure.^19^ In another study, Li and Xu have used supercritical water without a catalyst to evaluate and optimize recycling conditions for printed circuit boards composed of brominated epoxy resin. In that study, the authors decomposed the brominated epoxy resin and recovered 99.8% of the metals at 495 °C and 33 MPa pressure in 305 min.^23^ Although these researchers achieved their objectives, these methodologies require use of solvents at high temperatures and pressures. Other approaches employed by various researchers involve chemical degradation via acid or base reactions^24−26^ or use of a catalyst to decompose the epoxy resin.^22,27−29^ Some studies have involved a complete or partial decomposition of epoxy thermoset polymers and used the final products to create functional materials for several applications.^30−33^ For example, Tian et al. recycled thermoset epoxy resin using a microwave-assisted method to fabricate micro/nonporous materials that prove to have excellent performance in separation of oil/water emulsions.^30^ In another study, Liu et al. completely dissolved the thermoset epoxy resin using a combination of microwave-assisted swelling of the TER and nitric acid degradation. Then, these authors employed the decomposition products to dip coat a melamine foam that showed great performance for absorbing oils and separation of oil/water systems.^32^ However, these methodologies also present disadvantages by employing extreme oxidative conditions that could affect the environment and/or use of an expensive catalyst. Thus, a new, robust, and economically feasible recycling method for thermoset epoxies, which is considered environmentally friendly, sustainable, and economically viable and allows reuse of epoxy materials and carbon fibers with high-quality mechanical properties, is of increasing interest.

In the study reported herein, a liquid-phase recycling procedure employing ILs as green solvents is evaluated for recycling thermoset epoxies and their composites. ILs are defined as organic salts with melting points less than or equal to 100 °C and are typically composed of bulky cations with organic or inorganic counteranions.^34−37^ Various properties of ILs, such as low vapor pressure, high thermal stability, and high conductivity, make these solvents excellent candidates for use in epoxy recycling procedures. In this manuscript, epoxy dissolution and swelling in the presence of various alkyl-methyl-imidazolium ILs and alcohols were performed to determine the depolymerization capacity of these co-solvents under relatively mild conditions in the absence of catalysts. In addition, recovered epoxy monomers were employed for synthesis of a recycled epoxy resin, and its mechanical properties were further analyzed and compared with unused parent epoxy. Finally, carbon fibers were recovered with good properties using these optimal recycling conditions.

## Results and Discussion

### Synthesis and Characterization of ILs

Each alkyl-methyl-imidazolium IL was synthesized as described above. Figure S1 is a representative scheme for the synthesis of the two halide-containing ILs (1-hexyl- and 1-octyl-methylimidazolium). After this synthesis procedure, each IL was characterized using FT-IR, ^1^H NMR, and ESI. Results are presented in the Supporting Information (Figures S2–S7).

### Composite Characterization

**Figure 1:**
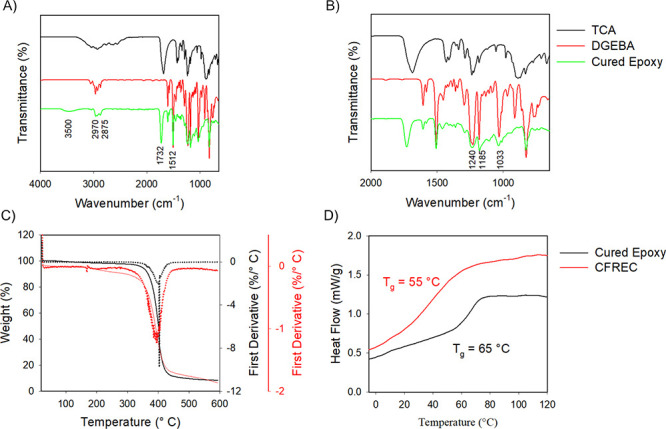
FT-IR spectra of each component of the thermoset epoxy and the cured thermoset in (A) the entire spectral region and (B) the fingerprint region. (C) TGA and (D) DSC curves of cured epoxy (black lines) and CFREC (red lines).

Synthesized epoxy resin was characterized through use of various analytical techniques such as FT-IR, DSC, and TGA. Figure 1 is a display of the FT-IR spectra of TCA, DGEBA, and cured epoxy resin. Based on the evaluation of the FT-IR spectra (Figure 1A), two characteristic bands can be observed in the TCA spectrum: a broad band center at 3000 cm^–1^ and a band at 1700 cm^–1^ corresponding to OH– and C=O stretching bands, respectively. The presence of a band at 1732 cm^–1^ and three bands at 1240, 1185, and 1033 cm^–1^ corresponds to the ester carbonyl stretch of the cured epoxy resin and indicates that the curing process occurred successfully. A band at 1512 cm^–1^ from the aromatic C–C stretch band is present in DGEBA and cured epoxy, which also demonstrates that the esterification reaction was successful and indicates the presence of an aromatic ring in the cured epoxy. Additionally, bands at 2970 and 2875 cm^–1^ corresponding to the C–H stretch are present in TCA and cured epoxy.

Figure 1B is a display of the fingerprint FT-IR spectral region of the two initial components of TER and cured epoxy resin. While the spectral region between 400 and 1500 cm^–1^, also known as the fingerprint region, contains several overlapping bands, differentiation among the peaks corresponding to certain bonds is complicated.^38^ Evaluation of the cured epoxy resin spectrum shows characteristic peaks of each starting material. Thus, it was concluded that the curing process was successful.

The glass transition temperatures (Tg) of TER and CFREC were determined using differential scanning calorimetry (DSC). In this regard, two cooling and heating cycles from −40 to 200 °C were performed on each sample. The second heating curve was used to determine the glass transition temperature. The software employed to calculate the Tg values was TA Instrument Universal Analysis 2000, and the resultant graphs are depicted in Figure 1D. The cured epoxy and CFREC displayed respective Tg values of 65 and 55 °C. Additionally, TER and CFREC thermal stability was evaluated between room temperature and 600 °C using a heating ramp of 10 °C/min. Onset decomposition temperatures were determined using the first derivative of TGA curves for both TER and CFREC, and respective values of 403.5 and 394.5 °C were obtained (Figure 1C). Both decomposition temperatures are higher than the temperatures employed during the swelling and dissolution studies, indicating that both TER and CFREC are stable and are not degraded at experimental temperatures.

### Swelling and Dissolution Studies

The primary objective of this study is to dissolve and depolymerize TER in the presence of ILs and/or alcohols under mild conditions without addition of catalysts. However, it is well established that this type of epoxy material has high chemical and solvent resistance.^1,2^ A study by Kuang et al. reported a dissolution mechanism of epoxy resin when submerged in ethylene glycol (EG), along with several catalysts.^29,39^ For this reason, EG (diol) and Gly (triol) were chosen as alcohols to evaluate the swelling of TER (Figure S8).

**Figure 2:**
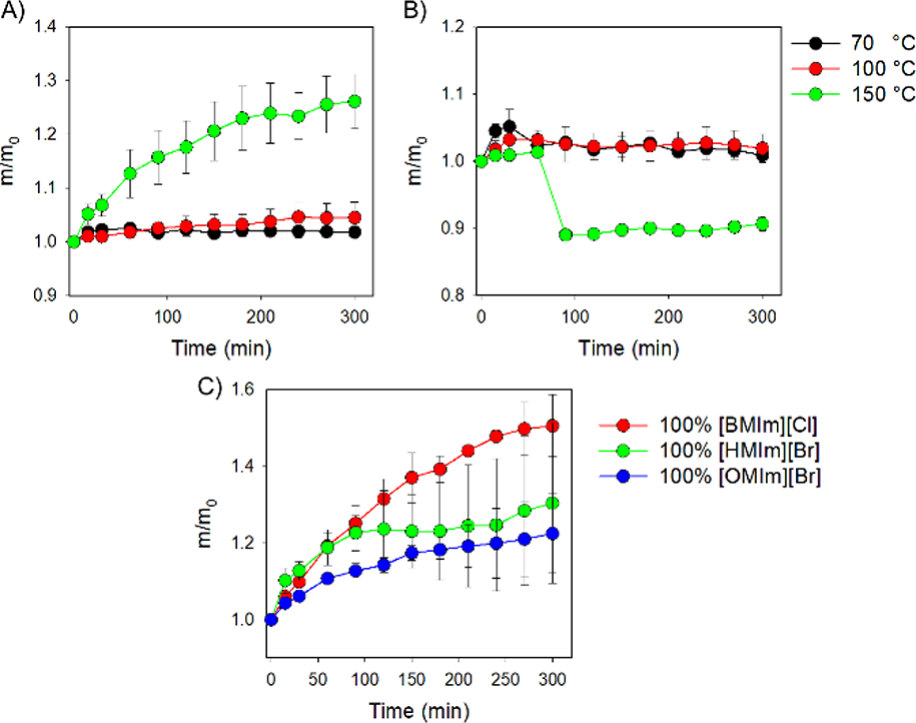
Normalized mass of TER over time at different temperatures in the presence of (A) EG and (B) Gly. (C) Normalized mass of TER over time in the presence of pure ILs.

Initially, the swelling of TER samples in the presence of EG and Gly was evaluated at three different temperatures: 70, 100, and 150 °C (Figure 2A,B). At higher temperatures, it is presumed that these molecules will have higher kinetic energy in solution and this will be reflected in increased intermolecular collisions.^40^ Evaluation of swelling results (Figure 2A,B) indicates that both alcohols used in this study were more effective at achieving epoxy interactions at higher temperatures. Moreover, a major swelling of the resin was observed at the highest temperature studied (150 °C) with EG, and a noticeable, although slight, decrease in epoxy mass was observed in the presence of Gly. We presume that this slight decrease in mass (9.35%) in the presence of Gly at 150 °C could be due to a larger hydroxy group to polymer ratio of these triol molecules against the external layer of the epoxy resin, which induces depolymerization through a transesterification reaction between the −OH group of the alcohol and the epoxy.^29^

According to the results presented in Figure 2A,B, the following swelling and dissolution experiments were performed at 150 °C, which are quite mild temperatures as compared to recent recycling literature studies.^19,41,42^ Following evaluation of the swelling process in the presence of pure alcohols EG and Gly, the swelling of TER was evaluated using pure alkyl-methyl-imidazolium ILs: [BMIm][Cl], [HMIm][Br], and [OMIm][Br] as solvents (Figure S9). Figure 2C is a display of the swelling experiments of TER samples in the presence of pure IL solvents with varying alkyl chains.

Evaluation of the results presented in Figure 2C demonstrated that all ILs studied are capable of penetrating the covalently cross-linked matrix of the epoxy thermoset. The largest increase in normalized mass was observed when TER used [BMIm][Cl]. In this case, [BMIm][Cl] swelled the thermoset polymer to 50% of the original mass as compared to the 30 and 22.5% swelling obtained with [HMIm][Br] and [OMIm][Br], respectively. This result is presumably in accordance with the increasing length of the alkyl chain in both ILs, as it was hypothesized that ILs with shorter chains would penetrate the polymer matrix in larger quantities.

**Figure 3:**
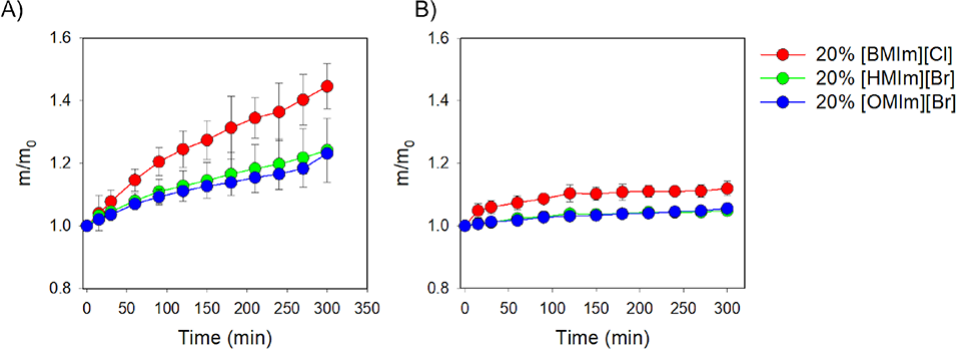
Normalized mass of epoxy thermoset over time in the presence of mixtures of 20% ILs plus (A) EG and (B) Gly.

The swelling and dissolution of the epoxy thermoset in the presence of different ratios of ILs and alcohols were then evaluated. Swelling experiments of TER were performed in the presence of each alcohol (EG or Gly) with three different ratios of 20, 40, and 60 weight % halide ILs. The first ratio studied was 20% IL to 80% alcohol (EG or Gly), and the results of these experiments are depicted in Figure 3. In this study, major swelling of TER was produced in the IL/EG solvent mixture when compared to the mixture of IL/Gly, which was in agreement with the results obtained in the presence of pure alcohols and pure ILs. The highest swelling of the cured epoxy thermoset, at 44.6%, was obtained in the presence of a 20:80 mixture of [BMIm][Cl]:EG. This mass increase was followed by swelling of approximately 27% in the presence of a [HMIm][Br] or [OMIm][Br] mixture with EG.

In contrast to the results presented in Figure 3B, mixtures of 20:80 IL:Gly swelled the TER at a lower magnitude as compared to the mixtures using diol. The highest swelling was observed in the presence of [BMIm][Cl], at approximately 12%, followed by [HMIm] and [OMIm] ILs, both with swelling capacities of approximately 5%.

The other ratios evaluated were 40 and 60% ILs (Figures S10 and S11). Analysis of these results that demonstrated similar trends in swelling results to the ratio 20:80 ILs:alcohol were obtained at the 40:60 and 60:40 ratios. A higher increment in epoxy mass of ∼40% was observed in the presence of [BMIm][Cl]:EG, and this result was in accordance with the results obtained using 20% ILs. For this reason, the following studies were performed using 20:80 ILs:EG mixtures. It is noted that more in-depth theoretical studies are desired to analyze the interaction between solvents and polymers. This includes investigating the plasticizing effect or swelling effect.^43−45^

### Swelling and Dissolution Experiments in Different Butyl-Methyl-Imidazolium ILs

Based on all ILs evaluated, the shortest alkyl chain imidazolium ([BMIm][Cl]) was determined to be the best for penetrating and swelling the cross-linked matrix of the TER at higher percentage. Although this co-solvent can swell the TER, it cannot dissolve the TER, making solvent-based recycling difficult. Therefore, different [BMIm]-based ILs with different anions were selected to further investigate the swelling and dissolution of TER. The anions acetate ([Acet^–^]), propionate ([Prop^–^]), hexanoate ([Hex^–^]), tetrafluoroborate ([BF4^–^]), and hexafluorophosphate ([PF6^–^]) were selected according to previously published reports that describe epoxy dissolution.^39,43^

**Figure 4:**
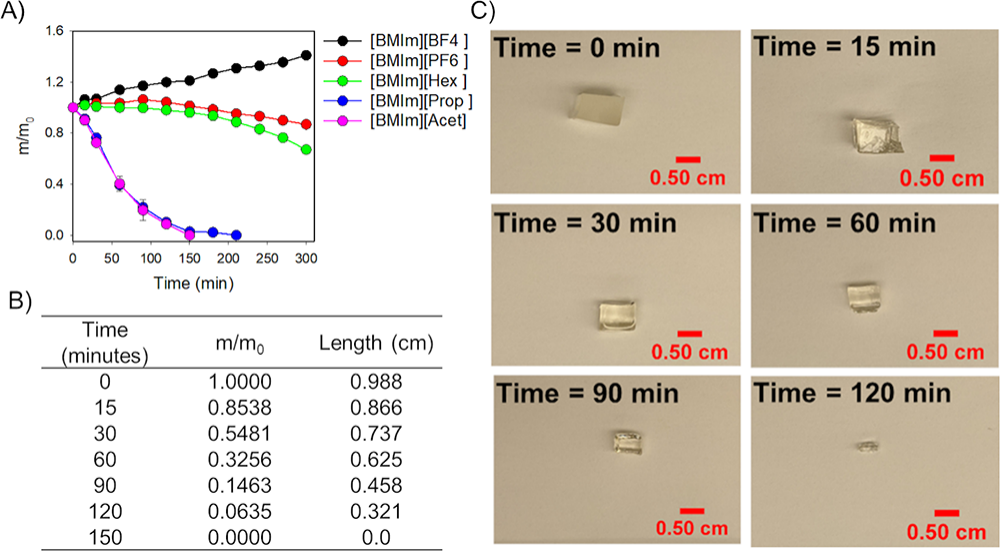
(A) Normalized mass of TER over time in the presence of mixtures of 20:80 of [BMIm]-based ILs and EG mixtures. (B) Normalized mass and length of the TER sample at different times when subjected to dissolution conditions with 20:80 [BMIm][Acet]:EG. (C) TER sample size and appearance at different times in the presence of 20:80 [BMIm][Acet]:EG solution at 150 °C.

Figure 4A is a display of the swelling experiments for TER in the presence of 20% [BMIm]-based ILs and 80% EG. It was observed that ILs with carboxylate anions completely dissolved the TER relative to all anions for ILs in this study. [BMIm][Acet] presented the best performance, by dissolving TER in only 150 min, followed by [Prop^–^] and [Hex^–^] anions. These results presented a similar trend to those observed with swelling experiments for different alkyl chains in the imidazolium ILs. In these experiments, [BMIm][Hex] with the longer alkyl chain carboxylate anion only dissolved approximately 33% of TER in the designated time frame. In contrast, [BMIm][Prop] dissolved the resin completely in 210 min. Figure 4C are pictures of TER at different time points with a 20:80 [BMIm][Acet]:EG solvent mixture. Analyses of these pictures along with the length and ratio of mass measured (Figure 4B) show the decreasing size of TER over time, until complete dissolution and disappearance at 150 min.

**Figure 5:**
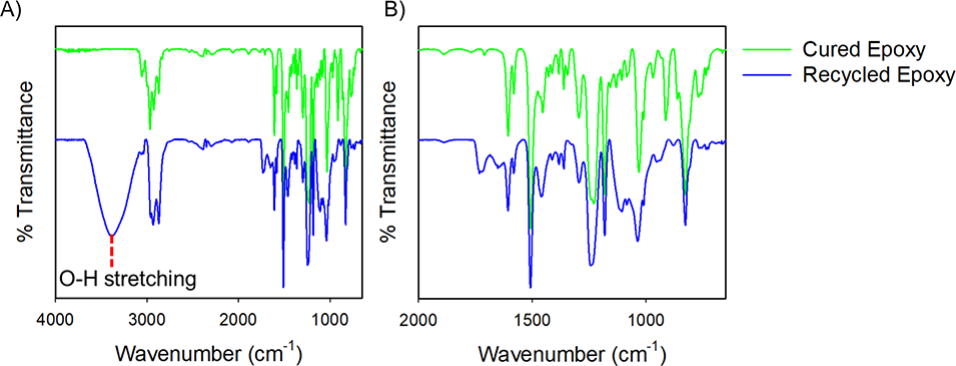
FT-IR spectra of cured epoxy and depolymerized epoxy resin (A) in the range of 4000–650 cm^–1^ and (B) fingerprint region.

We hypothesize that the mechanism of dissolution occurs via assistance from electrons present in oxygen of the carboxylate anion, which promotes attack on the polymeric ester bond. In the presence of the alcohol solvent, this allows generation of the alcohol derivative of the DGEBA molecule. This was verified through observation of the resultant −OH band in the FT-IR spectra of the recovered monomer (Figure 5). Additionally, ^1^H NMR experiments were performed with the DGEBA starting material and the depolymerized TER (Figure S12). Evaluation of results presented in Figure S12 indicates that the aromatic moieties of DGEBA remain unmodified during the recycling procedure. Some changes in the depolymerized TER spectrum were observed; however, further studies for structural elucidation as well as composition are required.

### Recovery of Recycled Epoxy and Carbon Fibers and Synthesis and Evaluation of New Thermoset Epoxy Resin and Carbon Reinforced Epoxy Composite

Once the TER has completely dissolved into the 20:80 [BMIm][Acet]:EG mixture, precipitation of the epoxy monomer was evaluated using several solvents such as acetonitrile, methanol, acetone, and water. Based on solvents studied, only water precipitated the depolymerized TER from solution. After precipitation, the solid was recovered using centrifugation and freeze drying to eliminate the remaining water.

**Figure 6:**
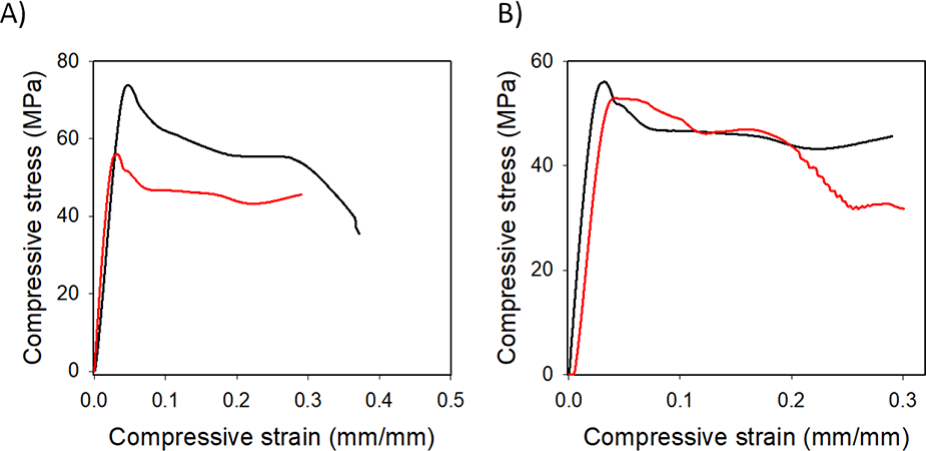
Compressive stress–strain behaviors of (A) new epoxy resin (black line) and recycled epoxy (red line) and (B) new CFREC (black line) and recycled CFREC (red line).

Five weight percent of depolymerized resin was then employed to synthesize new recycled TER. The physical characteristics of the new synthesized resin were similar to those obtained for 100 weight percent DGEBA from a commercial source. Figure 6A is a display of the compression mechanical experiments performed for both TERs. Based on analyses of these results, the compressive strength and Young’s modulus were calculated (Table 1). Evaluation of these results indicates that the recycled resin maintained similar mechanical properties as compared to the newly synthesized resin. Only an approximately 8% decrease in both Young’s modulus and compressive strength was observed in the recycled resin as compared to the new resin (Table 1).

**Table 1:** Mechanical Properties of New and Recycled Resin and CFREC

parameter	new resin	recycled resin
Young’s modulus (MPa)	2439 +/– 187	2251 +/– 187
compressive strength (MPa)	67 +/– 8	62 +/– 10

parameter	new CFREC	recycled CFREC
Young’s modulus (MPa)	2384 +/– 81	2299 +/– 42
compressive strength (MPa)	75 +/– 6	73 +/– 6

**Figure 7:**
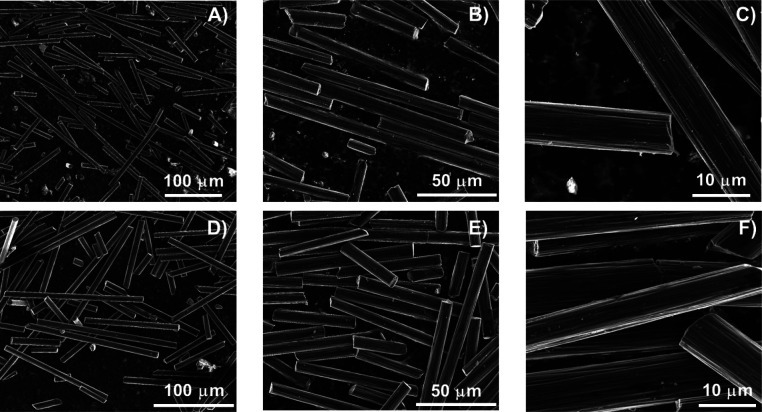
SEM micrographs of (A,B,C) new CF and (D,E,F) recycled CF.

Finally, recovery of CF from CFREC was evaluated. For this purpose, an approximately 3000 mg piece of CFREC was subjected to the optimal dissolution protocol in a solution of 20:80 [BMIm][Acet]:EG and placed in an oil bath at 150 °C for 150 min. Under these conditions, all CFREC was completely dissolved. The mixture of the depolymerized resin, CF, and 20:80 [BMIm][Acet]:EG was diluted with THF. The solution obtained was filtered in order to recover CF. After filtration, CF was washed with this solvent three times and recovered through centrifugation. Recovered CF was evaluated using SEM, and as observed in Figure 7, recovered milled carbon fibers presented smooth surfaces, which suggests that the resin was completely removed without affecting the structural integrity and morphology of CF.

Additionally, five weight percent of recovered CF was employed to synthesize recycled CFREC following the same procedure mentioned in the experimental section. After curing, the mechanical properties of recycled CFREC were evaluated and compared to those of the original CFREC (Figure 6B). Table 1 lists the Young’s modulus and compressive strength for both CFREC and recycled CFREC. Based on evaluation of data presented in Table 1, it was determined that compressive strength values decreased by approximately 2.7% and Young’s Modulus values dropped by less than 4% with respect to the original CFREC and the recycled CFREC.

### Evaluation of the Performance of the Recycled [BMIm][Acet]:EG Solvent Mixture after the TER Recycling Procedure

**Figure 8:**
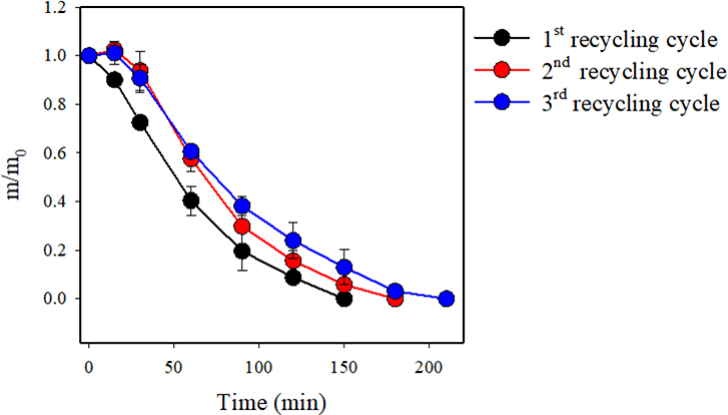
Normalized mass of TER over time in the presence of 20:80 [BMIm][Acet]:EG solvent mixtures at different recycling cycles.

After recovering the depolymerized TER through precipitation with water from the 20:80 [BMIm][Acet]:EG mixture, recycling of the IL:alcohol solvent mixture for subsequent TER depolymerization procedures was evaluated. The aqueous mixture [BMIm][Acet]:EG was frozen using liquid nitrogen, and water was removed through lyophilization. The recovered [BMIm][Acet]:EG solvent system was then employed in a subsequent recycling procedure following the same experimental conditions. Interestingly, the use of recovered 20:80 [BMIm][Acet]:EG in another cycle of TER recycling achieved the complete dissolution of the TER in 180 min, which is only 30 min longer than the initial TER recycling time (Figure 8). Furthermore, this 20:80% [BMIm][Acet]:EG mixture employed in a second TER recycling trial was recovered once more and used to recycle TER for a third time. Evaluation of results presented in Figure S13 indicate that although the twice recovered [BMIm][Acet]:EG mixture was able to completely dissolve and depolymerize the TER, this recycling procedure was 60 min longer than the first recycling procedure. This extended dissolution time could be a result of residual water and/or the result of partial decomposition of the IL:alcohol mixture, which could affect recycling capacity (Figure S13). However, analyses of results in Figure 8 indicate that the 20:80 [BMIm][Acet]:EG solvent mixture can be reused in several TER recycling procedures without the addition of fresh IL or alcohol to the solvent mixture.

## Conclusions

In conclusion, an ecologically friendly recycling method for both TER and CFRC samples in the presence of ILs and alcohols under mild working conditions (150 °C and atmospheric pressure) is reported in this study. Based on all ILs evaluated, [BMIm][Acet] in the presence of EG was determined to completely dissolve TER samples in approximately 150 min under specified working conditions. The recovered monomer from the depolymerized resin was obtained through precipitation and was employed for the synthesis of a recycled TER. Additionally, CF were recovered as well from CFREC employing the optimal recycling conditions. Mechanical properties of recycled TER and CFREC were also similar to those of the parent TER and CFREC, indicating that the recovered materials could be employed in the synthesis of new TER and CFREC. Additionally, the 20:80 [BMIm][Acet]:EG dissolution mixture was recovered and employed in three subsequent recycling procedures, achieving good results and complete dissolution and depolymerization of the TER for all cycles. The proposed recycling method herein is more cost-efficient than others presented in the literature.^29^ For example, in order to recycle 1 kg of epoxy resin, the cost of this method is approximately $1,250,000 in comparison to the cost of the method proposed by Kuang et al. of $2,200,000. In this regard, we have explored a robust method for recycling TER in a relatively efficient and viable manner to potentially aid in the decrease of epoxy waste and increase its reusability.

It is noted that several additional studies are highly desired. One area of study involves characterizing the molecular structure of the depolymerized epoxy resin to better understand the degradation mechanism. Another area of study involves investigating the effects of using a higher percentage of depolymerized resin in the recycled epoxy, evaluating the recycling performance of the method proposed here in recycling epoxy resins with higher glass transition temperatures, directly measuring the mechanical properties of the recycled carbon fiber, and analyzing the interaction between solvents and polymers, especially the plasticizing effect or swelling effect. These will be research topics for future studies.

## Materials and Methods

### Reagents

All reagents and solvents were used as purchased without further purification. Tricarballylic acid (TCA), 1-butyl-3-methyl imidazolium acetate ([BMIm][Acet]), 1-methyl imidazole, and 1-butyl-3- methyl imidazolium chloride ([BMIm][Cl]) were purchased from Sigma Aldrich (St. Louis, MO). Bisphenol A Dyglycidyl ether (DGEBA), tetrahydrofuran (THF), dichloromethane (DCM), ethylene glycol (EG), glycerol (Gly), 1-bromohexane, and 1-bromooctane were purchased from VWR (Radnor, PA). PX-35 milled carbon fibers (length = 150–200 μm, diameter = 7.2 μm, lineal resistivity = 0.0761 Ω/cm) were generously provided by Zoltek Company (Bridgeton, MO).

### Instrumentation

Fourier transform infrared (FT-IR) spectra were recorded using a Bruker Tensor 27 spectrometer equipped with a PIKE MIRacle single-bounce attenuated total reflectance (ATR) cell. Spectra were collected over the 4000–500 cm^–1^ region using 64 scans with a resolution of 4 cm^–1^. Differential scanning calorimetry (DSC) experiments were performed using a DSC Q100 (TA Instruments, New Castle, NJ). Thermogravimetric experiments were performed using a TA 550 Discovery Series instrument (TA Instruments, New Castle, NJ). Mechanical studies were performed using an Instron 5969 universal tensile system with a 50 kN cell and controlled with BlueHill 3 software. Scanning electron microscopy (SEM) experiments were performed using an FEI Quanta 3D FEG FIB/SEM. Triply deionized water was obtained employing an Aires High Purity Water System (Port Allen, LA).

### Synthesis of Epoxy Resin

**Figure 9:**
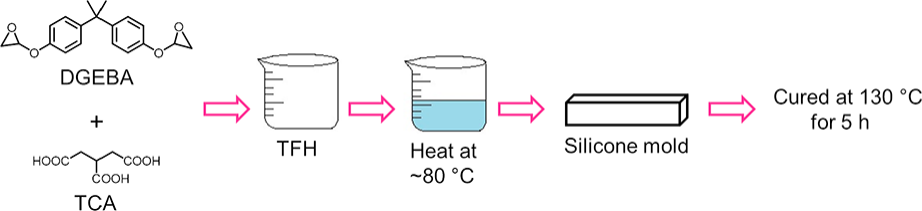
Scheme of the epoxy synthesis procedure.

Epoxy resin was synthesized according to a previously reported method.^44^ Briefly, DGEBA and TCA in a molar ratio of approximately 1 to 1 were dissolved in 40 mL of THF, stirred, and sonicated for 10 min. The esterification reaction was accelerated by placing the mixture in an oil bath at 80 °C and maintained at this temperature until all TCA was dissolved. When the solvent evaporated, the mixture was placed in a Teflon mold, and the epoxy was cured in an oven at 130 °C for 5 h. Finally, the oven was turned off, and the polymer was left inside to equilibrate overnight. Carbon fiber reinforced epoxy composites (CFRECs) were prepared in a similar manner. Five weight percent of milled fiber was added to a mixture of 1:1 molar ratio of DGEBA and TCA during the synthetic procedure. Figure S14 is a display of the initial materials of the epoxy resin. In Figure 9, a schematic representation of the epoxy synthetic method is displayed.

### Synthesis of ILs

Alkyl-methyl-imidazolium ILs were synthesized using the procedure described by Dzyuba and Bartsch^45^ Briefly, equimolar quantities of 1-methylimidazole and 1-bromo alkyl chains were stirred in a round-bottom flask at 140 °C for 10 min. The flask was then removed from the oil bath and allowed to cool for an additional 10 min. The flask was reintroduced into the oil bath at 140 °C for 10 min, and finally, the mixture was vacuum-dried at 100 °C for 2 h. Different [BMIm]-based ILs were synthesized through a metathesis reaction. [BMIm][Hex] and [BMIm][Prop] were synthesized by dissolving [BMIm][Cl] and the sodium salt of the corresponding anion in MeOH and allowed to stir for 48 h. The byproduct sodium chloride precipitated and was removed through filtration, and the IL was vacuum-dried.

Ionic liquids [BMIm][PF6] and [BMIm][BF4] were synthesized employing a procedure described by Cassol et al.^46^ Briefly, [BMIm][Cl] and [Na][BF4] or [K][PF6] in a molar ratio of 1:1.1 were placed in a vial, dissolved in triply distilled water, and stirred for 30 min. Each of the desired phases, the lower phase in the case of [BMIm][BF4] and the upper phase for [BMIm][PF6], were separated. In both cases, an excess of [Na][BF4] and [K][PF6] were added to the vials along with a small volume of triply distilled water and allowed to stir for an additional 15 min. An excess of DCM was added to extract the ILs from the aqueous layer, and the organic phase was separated, concentrated, and freeze-dried to remove residual water. All synthesized ILs were characterized using ^1^H NMR, ESI-MS, and FT-IR techniques, and the characterization results are provided in the Supporting Information.

### Swelling and Dissolution Experiments

Small rectangular pieces of TER (approximately 500 mg weight and 1 cm length by 0.5 cm width) were employed to prepare 5% W/W solutions in each pure solvent, either ILs or alcohols, or different ratios of IL-alcohol mixtures for evaluation. These solutions were placed in an oil bath at desired temperatures, and at different time points, the epoxy resin was removed from solution and weighed for a period of 300 min (5 h). All experiments were performed in triplicate. The swelling percentage was calculated by multiplying the final mass of TER or CFREC by 100% and dividing this value by the initial mass of TER or CFREC.

Monomers were recovered through precipitation with triply distilled water. Recycled epoxy resin was synthesized to contain 5 weight % of depolymerized monomer. The recycled monomer was dissolved in THF and mixed with 95 weight % DGEBA along with a curing agent. The curing procedure was performed as described in the preceding section.

## Supporting Information Available

Additional experimental results such as ^1^H NMR, FT-IR spectra of all synthetized compounds, reaction schemes, and structures (PDF)

## ABBREVIATIONS

[BMIm][Cl]: 1-butyl-3-methyl imidazolium chloride
[BMIm][Acet]: 1-butyl-3-methyl imidazolium acetate
[HMIm][Br]: 1-hexyl-3-methyl imidazolium bromide
[OMIm][Br]: 1-octyl-3-methyl imidazolium bromide
EG: ethylene glycol
Gly: glycerol
TER: thermoset epoxy resin
CFREC: carbon fiber reinforced epoxy composite
CFRP: carbon fiber reinforced polymer
DGEBA: Bisphenol A Dyglycidyl ether
THF: tetrahydrofuran
DCM: dichloromethane
FT-IR: Fourier transform infrared
DSC: differential scanning calorimetry

## References

[ref1] Jin F.-L.; Li X.; Park S.-J. Synthesis and application of epoxy resins: A review. J. Ind. Eng. Chem. 2015, 29, 1–11. 10.1016/j.jiec.2015.03.026.

[ref2] Nguyen H.; Zatar W.; Mutsuyoshi H. 4 - Mechanical properties of hybrid polymer composite, in Hybrid Polymer Composite Materials*;* Thakur V. K.; Thakur M. K.; Pappu A., Eds.; Woodhead Publishing; 2017; p. 83–113.

[ref3] May C. Epoxy resins: chemistry and technology; Routledge, 2018.

[ref4] Mohan P. A critical review: the modification, properties, and applications of epoxy resins. Polym.-Plast. Technol. Eng. 2013, 52, 107–125. 10.1080/03602559.2012.727057.

[ref5] Ou Y.; González C.; Vilatela J. J. Interlaminar toughening in structural carbon fiber/epoxy composites interleaved with carbon nanotube veils. Composites, Part A 2019, 124, 105477 10.1016/j.compositesa.2019.105477.

[ref6] Zanjani J. S. M.; Okan B. S.; Pappas P. N.; Galiotis C.; Menceloglu Y. Z.; Yildiz M. Tailoring viscoelastic response, self-heating and deicing properties of carbon-fiber reinforced epoxy composites by graphene modification. Composites, Part A 2018, 106, 1–10. 10.1016/j.compositesa.2017.12.008.

[ref7] Dong J.; Jia C.; Wang M.; Fang X.; Wei H.; Xie H.; Zhang T.; He J.; Jiang Z.; Huang Y. Improved mechanical properties of carbon fiber-reinforced epoxy composites by growing carbon black on carbon fiber surface. Compos. Sci. Technol. 2017, 149, 75–80. 10.1016/j.compscitech.2017.06.002.

[ref8] *CF & CFRP Market by Source (Virgin, Recycled), Precursor (PAN, Pitch, Rayon), Resin (Thermosetting, Thermoplastic), Manufacturing Process, End-use Industry, and Region - Global Forecast to 2025*.

[ref9] Ma C.; Sánchez-Rodríguez D.; Kamo T. Influence of thermal treatment on the properties of carbon fiber reinforced plastics under various conditions. Polym. Degrad. Stab. 2020, 178, 109199 10.1016/j.polymdegradstab.2020.109199.

[ref10] Pickering S. J. Recycling technologies for thermoset composite materials—current status. Composites, Part A 2006, 37, 1206–1215. 10.1016/j.compositesa.2005.05.030.

[ref11] Babu B. R.; Parande A. K.; Basha C. A. Electrical and electronic waste: a global environmental problem. Waste Manage. Res. 2007, 25, 307–318. 10.1177/0734242X07076941. 17874657

[ref12] Robinson B. H. E-waste: An assessment of global production and environmental impacts. Sci. Total Environ. 2009, 408, 183–191. 10.1016/j.scitotenv.2009.09.044. 19846207

[ref13] Kiddee P.; Naidu R.; Wong M. H. Electronic waste management approaches: An overview. Waste Manage. 2013, 33, 1237–1250. 10.1016/j.wasman.2013.01.006. 23402807

[ref14] Liu H.; Liang Y.; Zhang D.; Wang C.; Liang H.; Cai H. Impact of MSW landfill on the environmental contamination of phthalate esters. Waste Manage. 2010, 30, 1569–1576. 10.1016/j.wasman.2010.01.040. 20202809

[ref15] Rybicka J.; Tiwari A.; Leeke G. A. Technology readiness level assessment of composites recycling technologies. J. Cleaner Prod. 2016, 112, 1001–1012. 10.1016/j.jclepro.2015.08.104.

[ref16] Meyer L. O.; Schulte K.; Grove-Nielsen E. CFRP-Recycling Following a Pyrolysis Route: Process Optimization and Potentials. J. Compos. Mater. 2009, 43, 1121–1132. 10.1177/0021998308097737.

[ref17] Li H.; Englund K. Recycling of carbon fiber-reinforced thermoplastic composite wastes from the aerospace industry. J. Compos. Mater. 2017, 51, 1265–1273. 10.1177/0021998316671796.

[ref18] Pimenta S.; Pinho S. T. Recycling carbon fibre reinforced polymers for structural applications: Technology review and market outlook. Waste Manage. 2011, 31, 378–392. 10.1016/j.wasman.2010.09.019. 20980138

[ref19] Okajima I.; Hiramatsu M.; Shimamura Y.; Awaya T.; Sako T. Chemical recycling of carbon fiber reinforced plastic using supercritical methanol. J. Supercrit. Fluids 2014, 91, 68–76. 10.1016/j.supflu.2014.04.011.

[ref20] Liu Y.; Liu J.; Jiang Z.; Tang T. Chemical recycling of carbon fibre reinforced epoxy resin composites in subcritical water: Synergistic effect of phenol and KOH on the decomposition efficiency. Polym. Degrad. Stab. 2012, 97, 214–220. 10.1016/j.polymdegradstab.2011.12.028.

[ref21] Kim Y. N.; Kim Y.-O.; Kim S. Y.; Park M.; Yang B.; Kim J.; Jung Y. C. Application of supercritical water for green recycling of epoxy-based carbon fiber reinforced plastic. Compos. Sci. Technol. 2019, 173, 66–72. 10.1016/j.compscitech.2019.01.026.

[ref22] Keith M. J.; Román-Ramírez L. A.; Leeke G.; Ingram A. Recycling a carbon fibre reinforced polymer with a supercritical acetone/water solvent mixture: Comprehensive analysis of reaction kinetics. Polym. Degrad. Stab. 2019, 161, 225–234. 10.1016/j.polymdegradstab.2019.01.015.

[ref23] Li K.; Xu Z. Application of supercritical water to decompose brominated epoxy resin and environmental friendly recovery of metals from waste memory module. Environ. Sci. Technol. 2015, 49, 1761–1767. 10.1021/es504644b. 25582426

[ref24] Dang W.; Kubouchi M.; Sembokuya H.; Tsuda K. Chemical recycling of glass fiber reinforced epoxy resin cured with amine using nitric acid. Polymer 2005, 46, 1905–1912. 10.1016/j.polymer.2004.12.035.

[ref25] Hanaoka T.; Arao Y.; Kayaki Y.; Kuwata S.; Kubouchi M. Analysis of nitric acid decomposition of epoxy resin network structures for chemical recycling. Polym. Degrad. Stab. 2021, 186, 109537 10.1016/j.polymdegradstab.2021.109537.

[ref26] Ma Y.; Nutt S. Chemical treatment for recycling of amine/epoxy composites at atmospheric pressure. Polym. Degrad. Stab. 2018, 153, 307–317. 10.1016/j.polymdegradstab.2018.05.011.

[ref27] Liu T.; Zhang M.; Guo X.; Liu C.; Liu T.; Xin J.; Zhang J. Mild chemical recycling of aerospace fiber/epoxy composite wastes and utilization of the decomposed resin. Polym. Degrad. Stab. 2017, 139, 20–27. 10.1016/j.polymdegradstab.2017.03.017.

[ref28] Lo J. N.; Nutt S. R.; Williams T. J. Recycling benzoxazine–epoxy composites via catalytic oxidation. ACS Sustainable Chem. Eng. 2018, 6, 7227–7231. 10.1021/acssuschemeng.8b01790.

[ref29] Kuang X.; Zhou Y.; Shi Q.; Wang T.; Qi H. J. Recycling of Epoxy Thermoset and Composites via Good Solvent Assisted and Small Molecules Participated Exchange Reactions. ACS Sustainable Chem. Eng. 2018, 6, 9189–9197. 10.1021/acssuschemeng.8b01538.

[ref30] Tian F.; Yang Y.; Wang X.-L.; An W.-L.; Zhao X.; Xu S.; Wang Y.-Z. From waste epoxy resins to efficient oil/water separation materials via a microwave assisted pore-forming strategy. Mater. Horiz. 2019, 6, 1733–1739. 10.1039/C9MH00541B.

[ref31] Tian F.; Wang X.-l.; Yang Y.; An W.; Zhao X.; Xu S.; Wang Y.-Z. Energy-Efficient Conversion of Amine-Cured Epoxy Resins into Functional Chemicals Based on Swelling-Induced Nanopores. ACS Sustainable Chem. Eng. 2020, 8, 2226–2235. 10.1021/acssuschemeng.9b06013.

[ref32] Liu X.; Tian F.; Zhao X.; Du R.; Xu S.; Wang Y.-Z. Recycling waste epoxy resin as hydrophobic coating of melamine foam for high-efficiency oil absorption. Appl. Surf. Sci. 2020, 529, 147151 10.1016/j.apsusc.2020.147151.

[ref33] Liu X.; Tian F.; Zhao X.; Du R.; Xu S.; Wang Y.-Z. Multiple functional materials from crushing waste thermosetting resins. Mater. Horiz. 2021, 8, 234–243. 10.1039/D0MH01053G. 34821302

[ref34] Zhao Q.; Anderson J. L. 2.11 - Ionic Liquids. in Comprehensive Sampling and Sample Preparation; Pawliszyn J., Ed. Academic Press: Oxford, 2012: p. 213–242.

[ref35] Warner I. M.; El-Zahab B.; Siraj N. Perspectives on Moving Ionic Liquid Chemistry into the Solid Phase. Anal. Chem. 2014, 86, 7184–7191. 10.1021/ac501529m. 25017178

[ref36] Del Pópolo M. G.; Voth G. A. On the structure and dynamics of ionic liquids. J. Phys. Chem. B. 2004, 108, 1744–1752. 10.1021/jp0364699.

[ref37] Sun P.; Armstrong D. W. Ionic liquids in analytical chemistry. Anal. Chim. Acta 2010, 661, 1–16. 10.1016/j.aca.2009.12.007. 20113709

[ref38] Ismail A. A.; van de Voort F. R.; Sedman J. Chapter 4 Fourier transform infrared spectroscopy: Principles and applications*.* in Techniques and Instrumentation in Analytical Chemistry*;* Paré J. R. J.; Bélanger J. M. R. Eds.; Elsevier: 1997; p. 93–139.

[ref39] Kuang X.; Shi Q.; Zhou Y.; Zhao Z.; Wang T.; Qi H. J. Dissolution of epoxy thermosets via mild alcoholysis: the mechanism and kinetics study. RSC Adv. 2018, 8, 1493–1502. 10.1039/C7RA12787A. PMC9077052 35540886

[ref40] Perrin J. Brownian movement and molecular reality. 2013: Courier Corporation.

[ref41] Okajima I.; Sako T. Recycling fiber-reinforced plastic using supercritical acetone. Polym. Degrad. Stab. 2019, 163, 1–6. 10.1016/j.polymdegradstab.2019.02.018.

[ref42] Xu P.; Li J.; Ding J. Chemical recycling of carbon fibre/epoxy composites in a mixed solution of peroxide hydrogen and N,N-dimethylformamide. Compos. Sci. Technol. 2013, 82, 54–59. 10.1016/j.compscitech.2013.04.002.

[ref43] Maka H.; Spychaj T.; Pilawka R. Epoxy Resin/Ionic Liquid Systems: The Influence of Imidazolium Cation Size and Anion Type on Reactivity and Thermomechanical Properties. Ind. Eng. Chem. Res. 2012, 51, 5197–5206. 10.1021/ie202321j.

[ref44] Lu L.; Fan J.; Li G. Intrinsic healable and recyclable thermoset epoxy based on shape memory effect and transesterification reaction. Polymer 2016, 105, 10–18. 10.1016/j.polymer.2016.10.013.

[ref45] Dzyuba S. V.; Bartsch R. A. Efficient synthesis of 1-alkyl (aralkyl)-3-methyl (ethyl) imidazolium halides: Precursors for room-temperature ionic liquids. J. Heterocyclic Chem. 2001, 38, 265–268. 10.1002/jhet.5570380139.

[ref46] Cassol C. C.; Ebeling G.; Ferrera B.; Dupont J. A simple and practical method for the preparation and purity determination of halide-free imidazolium ionic liquids. Adv. Synth. Catal. 2006, 348, 243–248. 10.1002/adsc.200505295.

