# Supplementary material for: Recycling Thermoset Epoxy Resin Using Alkyl-Methyl-Imidazolium Ionic Liquids as Green Solvents
Source: ACS Appl Polym Mater. 2021 Oct 11;3(11):5588–95. doi: 10.1021/acsapm.1c00896 (PMC8593865; doi:10.1021/acsapm.1c00896)
Supplement: Supplementary file 1 — ap1c00896_si_001.pdf [file ap1c00896_si_001.pdf]

Supporting information

Recycling Thermoset Epoxy Resin using Alkyl-Methyl-Imidazolium Ionic Liquids as Green Solvents.

Rocío L. Pérez[a,*], Caitlan E. Ayala[a], Michelle M. Opiri[a], Abdulrahman Ezzir[a], Guoqiang Li[b], Isiah M. Warner[a,*]

[a] Department of Chemistry, Louisiana State University, Baton Rouge, LA, 70803

[b] Department of Mechanical & Industrial Engineering, Louisiana State University, Baton Rouge, LA, 70803

Corresponding authors: Isiah M. Warner, iwarner@lsu.edu; Rocío L. Pérez, rperez@lsu.edu, rperez@georgiasouthern.edu.

Synthesis and characterization of ILs:

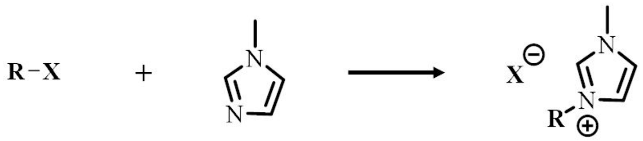

Figure S1: ILs synthesis scheme. R-groups employed in this reaction were hexyl and octyl alkyl chains.

1-hexyl, 3- methyl imidazolium bromide: ESI-MS: m/z expected: 167.15, m/z found: ^{1}H NMR ($CDCl_3$, 400 MHz, ppm): δ 10.22 (s, *J* = 1.0 Hz, 1H), 7.58 (s, *J* = 7.2 Hz, 1H), 7.41 (s, *J* = 5.6 Hz, 1H), 4.22 (s, *J* = 5.6 Hz, 2H), 4.04 (s, *J* = 7,2 Hz, 3H), 2.43 – 2.12 (m, *J* = 12,4 Hz, 1H), 1.83 (s, *J* = 5.6 Hz, 2H), 1.22 (s, *J* = = 5.6 Hz,6H), 0.78 (s, *J* = 7,2 Hz, 3H).

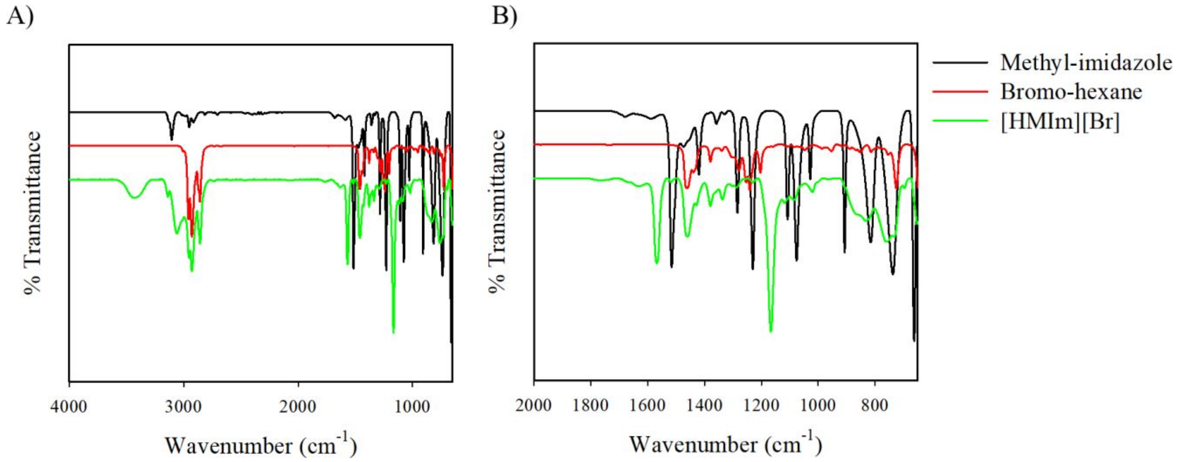

Figure S2: FT-IR spectra of 1-methylimidazole, 1-bromohexane and [HMIm][Br] in A) the entire spectral region, and B) the finger-print region.

1-octyl, 3- methyl imidazolium bromide: ESI: m/z expected: 195.15, m/z found: 195.19. ^{1}H NMR (DMSO, 400 MHz, ppm): δ 9.45 (s, 1H), 7.85 (s, 1H), 7.78 (s, 1H), 4.19 (t, *J* = 5.2 Hz, 2H), 3.87 (s, 3H), 1.77 (, *J* = 10.4 Hz, 2H), 1.24 (m, 10H), 0.84 (s, *J* = 6.8 Hz, 3H);

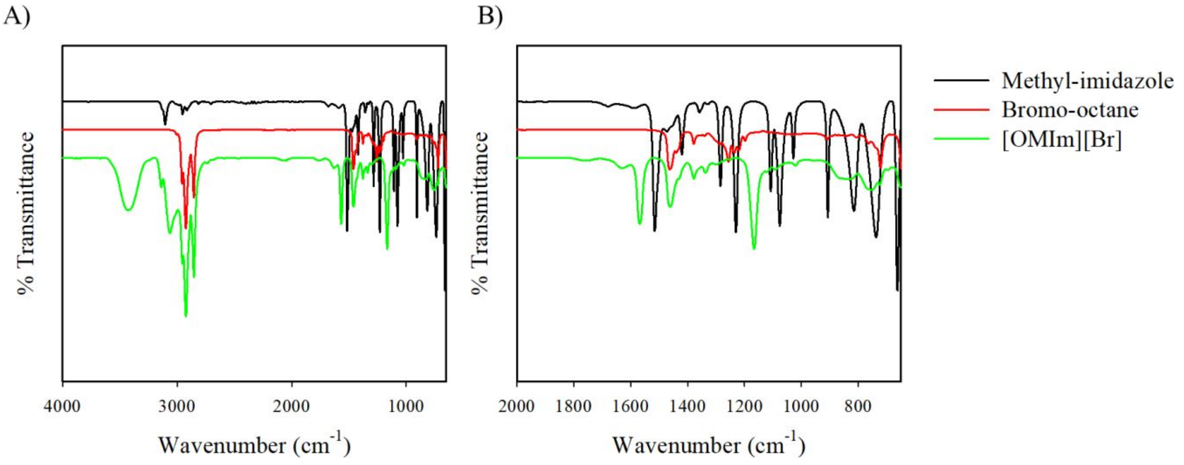

Figure S3: FT-IR spectra of 1-methyl-imidazole, 1-bromooctane and [OMIm][Br] in A) the entire spectral region, and B) the finger-print region.

1-butyl, 3-methyl imidazolium hexafluorophosphate: ESI: cation m/z expected: 139.12, cation m/z found: 139.12; anion m/z expected: 144.96, anion m/z found: 144.96.

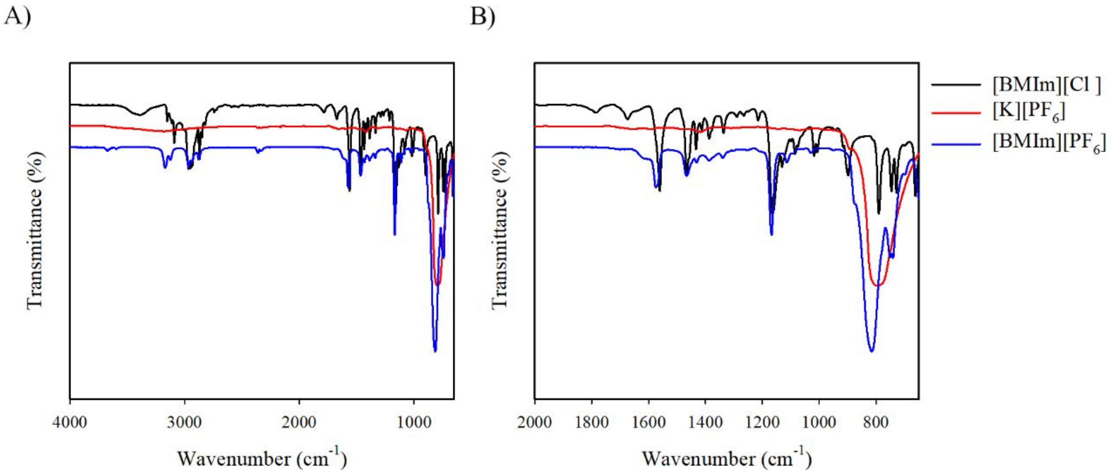

Figure S4: FT-IR spectra of [BMIm][Cl], [K][PF_6], and [BMIm][PF_6] in A) the entire spectral region, and B) the finger-print region.

1-butyl-3-methyl imidazolium tetrafluoroborate: ESI: cation m/z expected: 139.12, cation m/z found: 139.12; anion m/z expected: 87.00, anion m/z found: 87.00.

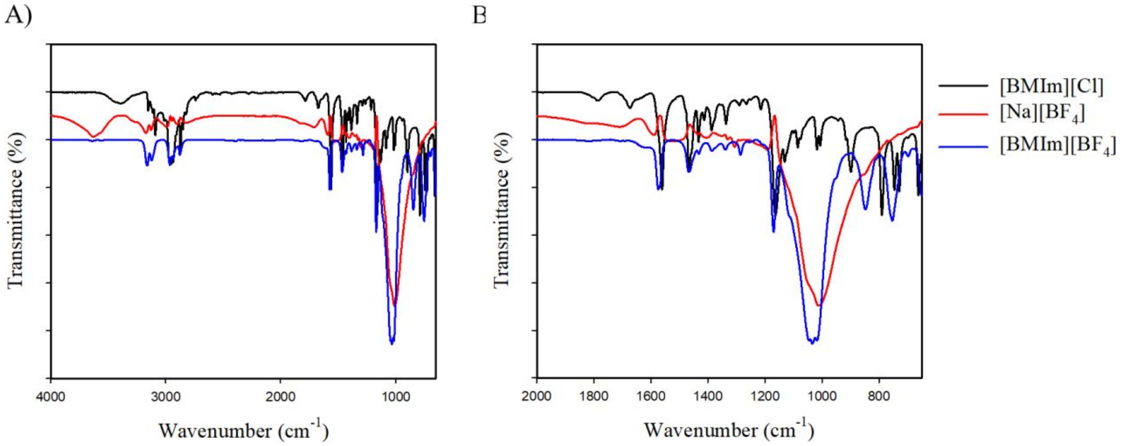

Figure S5: FT-IR spectra of [BMIm][Cl], [Na][BF_4], and [BMIm][BF_4] in A) the whole spectral region, and B) the finger-print spectral region.

1-butyl-3-methyl imidazolium propionate: ESI: cation m/z expected: 139.12, cation m/z found: 139.12. ^{1}H NMR (DMSO, 400 MHz, ppm): δ 9.49 (s, 1H), 7.81 (s, 1H), 7.75 (s, 1H), 4.19 (t, *J* = 6.0 Hz, 2H), 3.87 (s, 3H), 1.79 (m, *J* = 7.6 Hz,3H), 1.25 (m, *J* = 6.4 Hz,3H), 0.84 (m, *J* = 6.8 Hz, 6H);

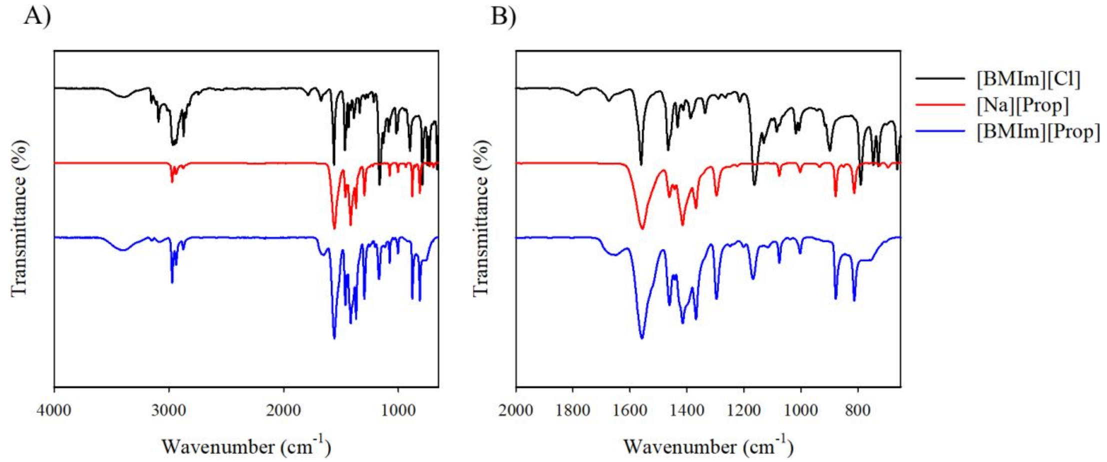

Figure S6: FT-IR spectra of [BMIm][Cl], [Na][Prop], and [BMIm][Prop] in A) the entire spectral region, and B) the finger-print region.

1-butyl, 3-methyl imidazolium hexanoate: ESI: cation m/z expected: 139.12, cation m/z found: 139.12; anion m/z expected: 115.08, anion m/z found: 115.07. ^{1}H NMR (DMSO, 400 MHz, ppm): δ 9.22 (s, 1H), 7.78 (s, 1H), 7.72 (s, 1H), 4.16 (t, *J* = 5.6 Hz, 2H), 3.85 (s, 3H), 3.35 (s, 3H), 1.79 (m, *J* = 9.6 Hz,3H), 1.39 (t, *J* = 5.6Hz, 2H), 1.25 (m, *J* = 7.2 Hz,4H), 0.93 (m, *J* = 8.8 Hz, 6H).

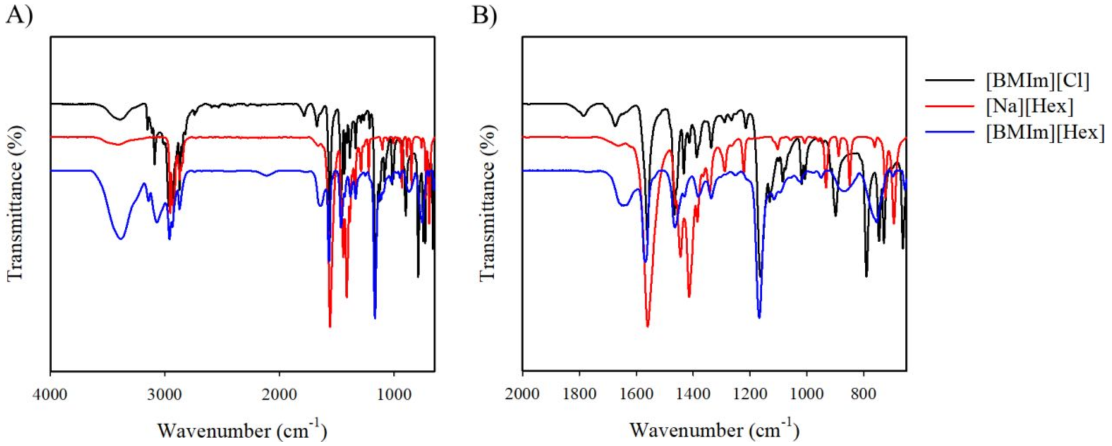

Figure S7: FT-IR spectra of [BMIm][Cl], [Na][Hex], and [BMIm][Hex] in A) the entire spectral region, and B) the finger-print region.

Swelling and dissolution studies

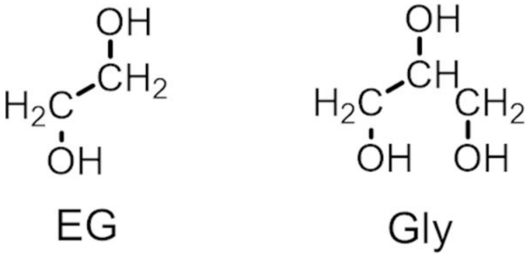

Figure S8: Chemical structures of studied alcohols.

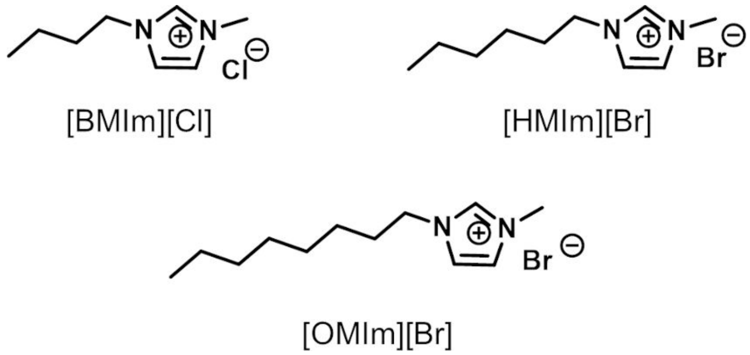

Figure S9: Chemical structures of studied ILs.

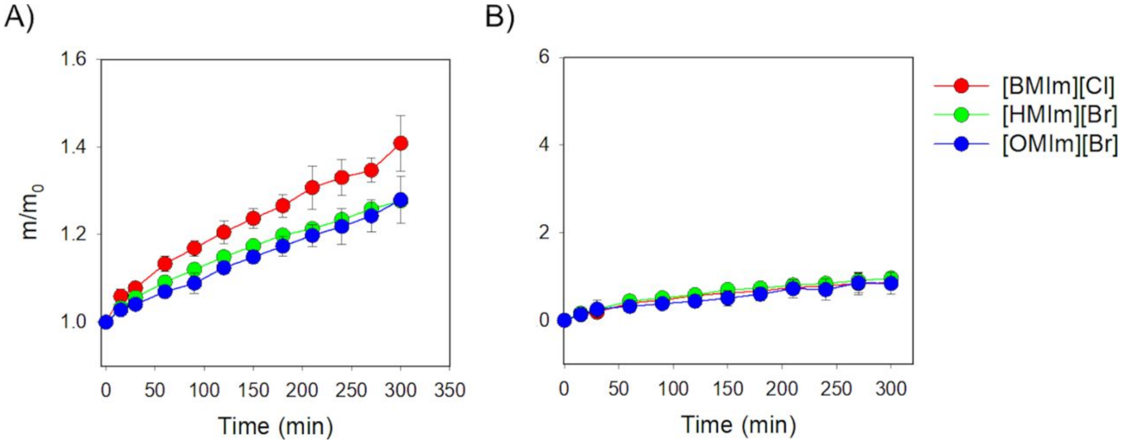

Figure S10: Normalized mass of epoxy thermoset over time in the presence of mixtures of 40% of ILs with A) EG or B) Gly.

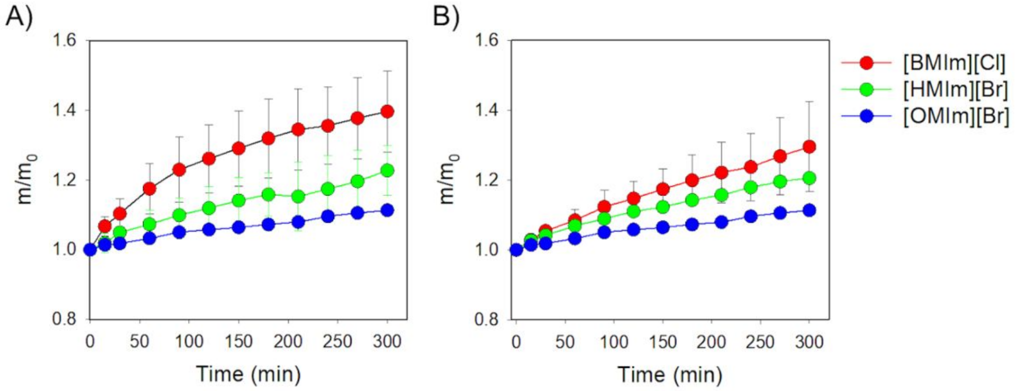

Figure S11: Normalized mass of epoxy thermoset over time in presence of mixtures of 60% of ILs with A) EG or B) Gly.

Recovery of recycled epoxy and carbon fibers, synthesis and evaluation of new thermoset epoxy resin and carbon reinforced epoxy composite

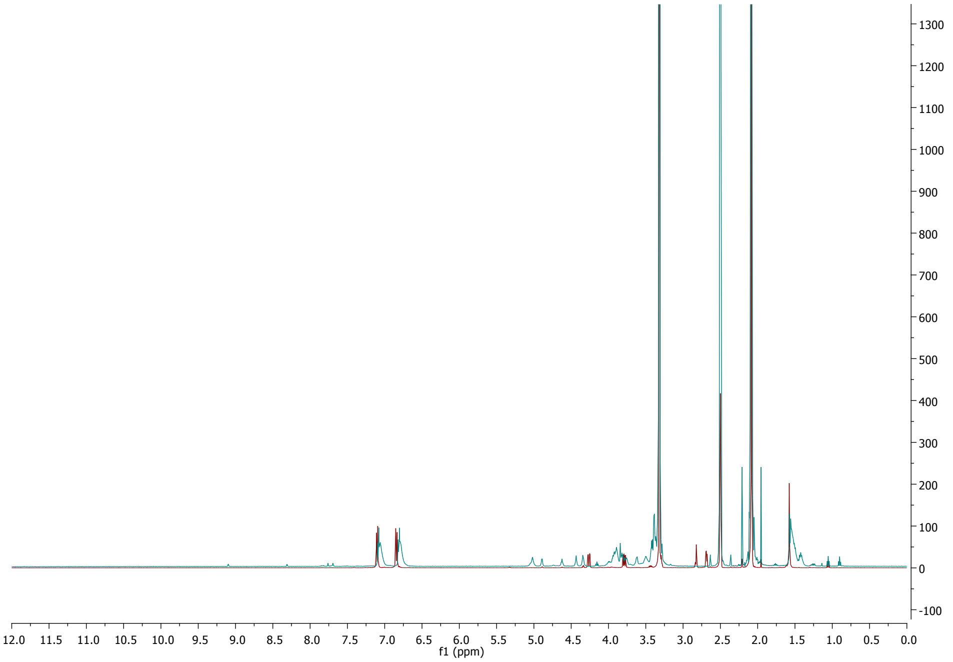

Figure S12: Proton (^{1}H) NMR spectra of DGEBA (red line), and depolymerized TER (green line) in d^6-DMSO.

Evaluation of performance of recycled [BMIm][Acet]:EG solvent mixture after TER recycling procedure

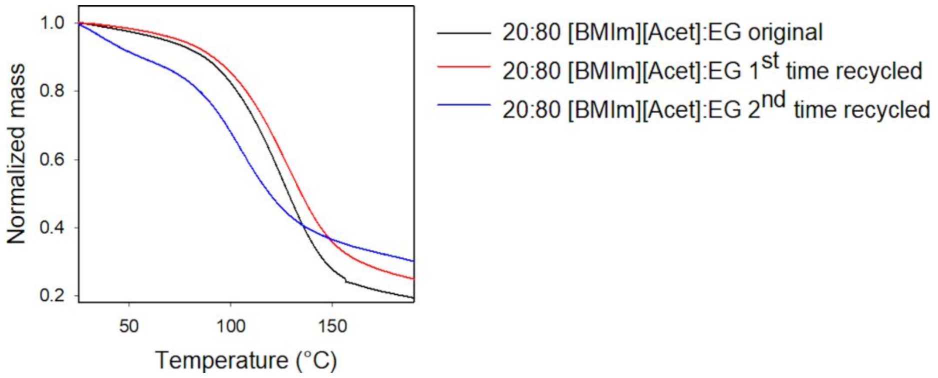

Figure S13: Thermogravimetric analysis of 20:80 [BMIm][Acet]:EG solvent mixtures.

Synthesis of epoxy resin:

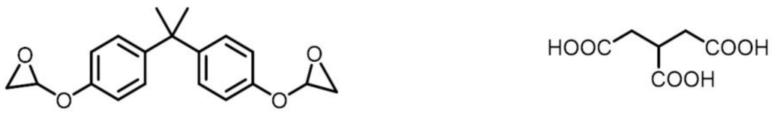

Diglycidyl Ether of Bisphenol A (DGEBA) Tricarballylic Acid (TCA)

Figure S14: Chemical structures of starting materials and epoxy polymer
